# Strategies and innovations in hypertension management for sickle cell patients: A narrative review

**DOI:** 10.1097/MD.0000000000047703

**Published:** 2026-02-13

**Authors:** Emmanuel Ifeanyi Obeagu

**Affiliations:** a Division of Haematology, Department of Biomedical and Laboratory Science, Africa University, Mutare, Zimbabwe; b Department of Molecular Medicine and Haematology, School of Pathology, Faculty of Health Sciences, University of the Witwatersrand, Johannesburg, South Africa.

**Keywords:** hypertension, innovations, management, sickle cell disease, strategies

## Abstract

Sickle cell disease (SCD) remains a challenging hematologic disorder, characterized by chronic hemolysis, vaso-occlusive events, and multi-organ complications. Hypertension, a prevalent comorbidity in SCD, poses significant clinical implications, exacerbating the complexities of disease management and impacting patient outcomes. Understanding the intricate interplay between SCD and hypertension is pivotal. Mechanistic insights uncover a landscape characterized by chronic hemolysis, endothelial dysfunction, altered nitric oxide bioavailability, and increased oxidative stress, contributing to elevated blood pressure and heightened cardiovascular risks in individuals with SCD. The diagnostic challenges inherent in identifying and monitoring hypertension in SCD patients necessitate novel approaches. Current treatment paradigms encompass a spectrum of lifestyle modifications, pharmacological interventions, and multidisciplinary care models. However, the limitations and complexities inherent in managing hypertension in SCD call for innovative strategies. Tailored approaches, personalized treatments, and emerging therapeutic avenues geared explicitly toward SCD patients mark a shift toward more effective management. Advancements in technology, including wearable devices and remote monitoring systems, present opportunities to revolutionize blood pressure monitoring, enhancing patient engagement and compliance while providing more accurate and frequent measurements. Moreover, the review underscores the importance of integrated care models and multidisciplinary collaborations. Collaborative frameworks involving hematologists, cardiologists, nephrologists, and primary care physicians are integral in optimizing hypertension management and addressing the specific needs of individuals with SCD.

## 1. Introduction

Hypertension poses a significant health concern for individuals living with sickle cell disease (SCD), presenting unique challenges in management and treatment.^[1]^ SCD, a genetic blood disorder characterized by abnormal hemoglobin, not only predisposes individuals to vaso-occlusive crises but also increases the risk of developing hypertension.^[2]^ This dual burden places patients at heightened risk of complications, necessitating tailored strategies and innovative approaches for effective hypertension management within the SCD population. Individuals with SCD often experience endothelial dysfunction, chronic inflammation, and increased oxidative stress, contributing to the pathogenesis of hypertension.^[3]^ Moreover, the chronic anemia associated with SCD can stimulate compensatory mechanisms, such as increased cardiac output and peripheral vascular resistance, further exacerbating hypertension.^[4]^ Consequently, interventions targeting both the underlying mechanisms of SCD and hypertension are essential for optimal patient outcomes.

In recent years, there has been a growing recognition of the need for personalized and multidisciplinary approaches to hypertension management in SCD patients. Integrating hematology, cardiology, nephrology, and primary care services allows for holistic assessment and management of both SCD-related complications and hypertension. Furthermore, advancements in technology, such as telemedicine and remote monitoring devices, have facilitated greater accessibility to specialized care, particularly for individuals residing in remote or underserved areas. Pharmacological interventions remain a cornerstone in the management of hypertension in SCD patients, yet selecting appropriate antihypertensive agents poses unique challenges.^[5]^ Certain medications commonly used in the general population, such as angiotensin-converting enzyme (ACE) inhibitors and angiotensin II receptor blockers (ARBs), may have limited efficacy or safety concerns in individuals with SCD due to their effects on renal function and intravascular volume. Consequently, there is a growing interest in exploring alternative pharmacotherapies tailored to the pathophysiology of both SCD and hypertension. In addition to pharmacotherapy, lifestyle modifications play a pivotal role in hypertension management among SCD patients.^[6]^ Dietary interventions, including sodium restriction and adherence to the Dietary Approaches to Stop Hypertension diet, can help mitigate hypertension risk factors while promoting overall cardiovascular health. Furthermore, regular physical activity tailored to individual capabilities can aid in blood pressure (BP) control and improve endothelial function, thereby reducing the burden of hypertension in this population. Beyond conventional approaches, emerging therapeutic modalities hold promise for revolutionizing hypertension management in SCD patients. Gene therapy, for instance, offers the potential to target underlying genetic mutations contributing to both SCD and hypertension, providing a more targeted and curative approach.^[7]^ Additionally, novel pharmacological agents targeting specific pathways implicated in SCD-related hypertension, such as endothelin receptor antagonists or nitric oxide (NO) donors, are currently under investigation and may offer new avenues for treatment.

## 2. Aim

The aim of this comprehensive review is to explore strategies and innovations in hypertension management specifically tailored for individuals living with SCD.

### 2.1. Rationale

The rationale for focusing on strategies and innovations in hypertension management for individuals with SCD stems from the recognition of the significant health burden posed by this dual condition. SCD is associated with a myriad of complications, including chronic anemia, vaso-occlusive crises, and endothelial dysfunction, all of which contribute to an increased risk of developing hypertension. This co-occurrence of SCD and hypertension presents unique challenges in management, as conventional approaches may be less effective or carry safety concerns in this population. Moreover, hypertension in SCD patients is not only associated with an elevated risk of cardiovascular complications but also exacerbates the progression of SCD-related organ damage, including renal dysfunction and pulmonary hypertension. Therefore, addressing hypertension in SCD patients is not only crucial for cardiovascular health but also for mitigating the overall disease burden and improving quality of life. Furthermore, the existing literature indicates a gap in tailored approaches to hypertension management in SCD patients, with limited guidance on optimal pharmacological therapies, and emerging therapeutic modalities. By addressing this gap, healthcare providers can better meet the unique needs of SCD patients, leading to improved patient outcomes and reduced healthcare disparities within this population. Additionally, with advancements in technology and therapeutic modalities, there exists an opportunity to innovate and optimize hypertension management in SCD patients. Exploring novel approaches, such as gene therapy or targeted pharmacotherapies, holds promise for revolutionizing treatment paradigms and addressing the underlying pathophysiology of both SCD and hypertension.

## 3. Review methodology

### 3.1. Literature search strategy

A systematic literature search was conducted using electronic databases including PubMed/MEDLINE, Scopus, Web of Science, and Google Scholar. Keywords and Medical Subject Headings terms related to SCD, hypertension, management, treatment, strategies, innovations, and interventions were utilized to identify relevant studies published from inception to the present.

### 3.2. Inclusion and exclusion criteria

Studies were included if they met the following criteria:

1. Published in peer-reviewed journals.
2. Written in English.
3. Investigated strategies, innovations, or interventions for hypertension management in individuals with SCD.
4. Included human subjects of all ages.

Studies were excluded if they:

1. Were conference abstracts, case reports, or editorials.
2. Did not focus on hypertension management in the context of SCD.
3. Were duplicates or unavailable in full text.

### 3.3. Data extraction

Data extraction was performed independently by 2 reviewers using a standardized data extraction form. Extracted information included study characteristics (author, year, study design), participant characteristics (sample size, demographics), interventions or strategies evaluated, outcomes measured, and key findings.

### 3.4. Quality assessment

The quality of included studies was assessed using appropriate tools depending on study design. Randomized controlled trials were assessed using the Cochrane Collaboration’s Risk of Bias Tool, while observational studies were evaluated using the Newcastle–Ottawa Scale for cohort and case–control studies.

### 3.5. Data synthesis

Findings from the included studies were synthesized narratively, categorizing interventions and strategies into pharmacological, and emerging therapeutic modalities. Key themes and trends across studies were identified, and gaps in the literature were discussed.

### 3.6. Ethical approval

Not applicable as this is a narrative review.

### 3.7. Understanding hypertension in sickle cell anemia (SCA)

Hypertension, commonly known as high BP, is a significant comorbidity that can affect individuals with SCA, a form of SCD.^[8]^ Hypertension is more prevalent in individuals with SCA compared to the general population.^[9]^ Factors contributing to its development in SCA patients include chronic anemia, impaired NO availability due to endothelial dysfunction, oxidative stress, and kidney damage associated with sickle cell nephropathy. The pathophysiological mechanisms linking SCA and hypertension are multifaceted. Chronic hemolysis, where red blood cells are destroyed at a higher rate than normal, contributes to endothelial dysfunction and decreased NO availability, leading to increased vascular resistance and potential elevation in BP. The presence of hypertension in individuals with SCA exacerbates the risk of cardiovascular complications.^[10]^ It can lead to increased risks of stroke, heart failure, renal impairment, and other end-organ damage. Additionally, hypertension in SCA patients may influence disease severity and prognosis. Diagnosing hypertension in individuals with SCA can be challenging due to overlapping symptoms and the potential impact of anemia on BP readings.^[1]^ Moreover, managing hypertension in the context of SCA requires careful consideration due to potential interactions between medications used for SCA treatment and those for hypertension. The management of hypertension in SCA typically involves lifestyle modifications such as adopting a low-sodium diet, regular exercise, and weight management.^[11]^ Pharmacological interventions, including antihypertensive medications, may be necessary, but medication choice and dosage adjustments should be tailored to the individual patient’s needs and specific health considerations (Table 1).

**Table 1:** Pathophysiologic mechanisms of hypertension in sickle cell disease.

Mechanism	Key features	Clinical implications
Endothelial dysfunction	Free hemoglobin scavenges nitric oxide (NO), reducing vasodilation.	Increased vascular tone, higher risk of pulmonary hypertension.
Renal pathology (nephropathy)	Glomerular hyperfiltration, proteinuria, altered sodium handling.	Volume overload, progressive chronic kidney disease.
Inflammation and oxidative stress	Elevated cytokines (IL-6, TNF-α), reactive oxygen species.	Vascular stiffness, sympathetic activation.
Autonomic dysregulation	Impaired baroreceptor sensitivity.	Blood pressure variability, difficulty in achieving stable control.
Hemolysis-associated vasoactivity	Release of arginase and free radicals.	Depletion of NO precursors, sustained vasoconstriction.

### 3.8. Challenges and diagnostic considerations

In the context of SCA, diagnosing and managing hypertension present several challenges and diagnostic considerations due to the unique physiological characteristics and complications associated with this condition.^[12]^ SCA patients often experience symptoms that can mimic or overlap with those of hypertension. Pain crises, fatigue, and respiratory issues may confound the assessment of hypertension, leading to challenges in accurately diagnosing elevated BP. Chronic anemia, a hallmark of SCA, can affect BP readings. Anemia-related factors, such as reduced viscosity and altered vascular tone, may result in lower BP measurements, potentially masking the presence of hypertension. Determining diagnostic thresholds for hypertension in SCA patients can be complex.^[13]^ Standard BP guidelines may need adjustment to account for the unique hemodynamic alterations and variability in BP readings often observed in individuals with SCA. Sickle cell nephropathy, a common complication of SCA, can contribute to the development of hypertension due to kidney damage.^[14]^ Distinguishing between hypertension as a primary condition or secondary to renal impairment is crucial for effective management. Obtaining accurate BP measurements in individuals with SCA poses challenges due to factors such as vaso-occlusive crises, pain episodes, or difficulty in cuff placement caused by arm deformities.^[15]^ Using appropriate cuff sizes and ensuring a relaxed and pain-free environment during measurements are critical. Differentiating between symptoms of hypertension and those attributed to other SCA-related complications can be intricate. Pain crises or symptoms related to anemia might mask or mislead clinicians in identifying hypertension. Long-term monitoring of BP in SCA patients can be challenging due to variability in readings, especially during acute sickle cell-related events.^[16]^ The reliability of BP measurements in such circumstances remains a concern for accurate diagnosis and management.

### 3.9. Current treatment landscape

The treatment landscape for hypertension in individuals with SCA involves a multifaceted approach that integrates pharmacological interventions, and individualized care plans.^[17]^ Lifestyle changes form the cornerstone of managing hypertension in SCA.^[18]^ Recommendations include adopting a heart-healthy diet with reduced sodium intake, increasing consumption of fruits and vegetables, regular physical activity, maintaining a healthy weight, and avoiding smoking and excessive alcohol intake. These modifications aim to control BP and reduce cardiovascular risks. Pharmacotherapy is often necessary for effectively controlling BP in individuals with SCA.^[19]^ Antihypertensive medications such as calcium channel blockers, ACE inhibitors, ARBs, beta-blockers, or diuretics may be prescribed based on individual patient needs. However, the choice of medication and dosage adjustments should consider potential interactions with SCA-related treatments and the patient’s specific health status.

Tailoring treatment plans according to the unique characteristics and needs of each SCA patient is essential.^[20]^ Personalizing therapy involves considering factors such as age, disease severity, presence of other SCA-related complications, and potential interactions between medications used for managing SCA and those for controlling hypertension. Regular monitoring of BP is crucial to assess treatment effectiveness and adjust medication dosages as needed. Frequent follow-up appointments with healthcare providers enable ongoing evaluation, optimization of treatment regimens, and early detection of potential adverse effects or complications. Addressing complications specific to SCA, such as pain crises, anemia, or sickle cell nephropathy, indirectly influences BP control.^[21]^ Effective management of these complications can mitigate physiological stressors that contribute to hypertension. Encouraging patient adherence to prescribed medications is essential for successful management. Patient education, regular counseling, and support systems play pivotal roles in promoting adherence and achieving better BP control. The treatment landscape for hypertension in individuals with SCA emphasizes a holistic approach that integrates, appropriate pharmacotherapy, individualized care plans, and close monitoring. Tailored management strategies considering the complexities of both conditions are vital to mitigate the risks associated with hypertension and improve overall health outcomes in individuals living with SCA. Emerging research indicates that sex and gender may significantly influence cardiovascular outcomes in patients with SCD. For instance, female patients may exhibit more favorable endothelial function and different responses to ACE inhibitors, possibly due to hormonal variations. Moreover, gender-based disparities in access to health services may delay hypertension diagnosis and management in women, particularly in resource-limited settings.

### 3.10. Innovations in hypertension management

Innovations in hypertension management continue to evolve, offering promising avenues for addressing the complexities of treating elevated BP, particularly in individuals with SCA.^[22]^ These innovations encompass technological advancements, personalized treatment approaches, and novel therapeutic strategies designed to improve monitoring, enhance treatment efficacy, and optimize patient outcomes. Wearable devices, such as smartwatches or fitness trackers equipped with BP monitoring capabilities, offer continuous and real-time tracking of BP trends. These devices provide convenience and enable individuals with SCA to monitor their BP regularly, facilitating early detection of fluctuations or abnormalities. Telemedicine platforms and remote monitoring systems enable healthcare providers to remotely assess BP readings and provide guidance or adjustments in treatment plans without the need for frequent in-person visits.^[23]^ This approach enhances accessibility to care, particularly for patients with chronic conditions like SCA and hypertension.

Advancements in pharmacogenomics allow for tailored treatment approaches based on an individual’s genetic profile. Understanding genetic variations affecting drug metabolism and response helps in optimizing medication selection and dosages, potentially improving treatment efficacy while minimizing adverse effects in SCA patients with hypertension. Ongoing research explores targeted therapies specifically designed to address the mechanisms underlying hypertension in SCA. Novel drug interventions targeting endothelial dysfunction, oxidative stress, or NO availability aim to provide more effective and targeted treatments for BP control in this population. Integration of machine learning algorithms and predictive analytics into healthcare systems enables better risk stratification, early identification of individuals at higher risk of developing hypertension complications, and personalized interventions.^[24]^ These technologies aid in improving diagnostic accuracy and treatment outcomes in SCA patients with hypertension. Mobile applications designed for patient education, medication adherence, play a significant role in engaging individuals in their own care.^[25]^ These apps offer personalized guidance, reminders for medications, dietary recommendations, and encourage healthy lifestyle behaviors, contributing to better BP control. Innovations in hypertension management, driven by technological advancements and personalized approaches, offer promising opportunities to enhance the diagnosis, monitoring, and treatment of elevated BP in individuals with SCA. These innovations strive to improve patient outcomes, optimize care delivery, and ultimately mitigate the associated risks and complications of hypertension in the context of SCA (Table 2).

**Table 2:** Strategies and innovations in hypertension management for sickle cell patients.

Intervention/strategy	Mechanism of action/benefit	Clinical considerations
ACE inhibitors/ARBs	Reduce intraglomerular pressure, protect renal function.	First-line for patients with proteinuria or microalbuminuria.
Calcium channel blockers (CCBs)	Promote vasodilation, lower systemic vascular resistance.	Monitor for peripheral edema; useful in concurrent pulmonary hypertension.
Beta-blockers	Decrease heart rate and cardiac output.	Caution in patients with severe anemia or reactive airway disease.
Diuretics (thiazide/loop)	Reduce volume overload.	Monitor electrolytes and renal function.
Hydroxyurea	Decreases hemolysis, improves NO bioavailability.	Indirect BP benefit via improved vascular function.
Endothelin receptor antagonists	Block endothelin-1–mediated vasoconstriction.	Emerging therapy; mainly studied for pulmonary hypertension.
Nitric oxide donors/arginine supplementation	Restore NO signaling, enhance vasodilation.	Investigational; dosing and long-term safety require further study.
Gene therapy/CRISPR-based interventions	Correct underlying hemoglobinopathy to reduce vascular dysfunction.	Still experimental; may provide long-term BP normalization.
Lifestyle interventions	Sodium restriction, tailored exercise, smoking cessation.	Requires careful balance to prevent dehydration or vaso-occlusive crises.
Telemedicine & remote monitoring	Improve adherence and early detection of BP changes.	Particularly valuable in low-resource settings.

ACE = angiotensin-converting enzyme, ARB = angiotensin II receptor blocker.

### 3.11. Integrated care models and multidisciplinary approaches

Integrated care models and multidisciplinary approaches play a pivotal role in optimizing the management of hypertension in individuals with SCA.^[26]^ These models emphasize collaborative efforts among healthcare professionals from various specialties to provide comprehensive and coordinated care tailored to the unique needs of SCA patients with hypertension. Integrated care models bring together a team of healthcare providers, including hematologists, cardiologists, nephrologists, primary care physicians, nurses, and other specialists, fostering collaboration and communication. This framework ensures a holistic approach to patient care, addressing both SCA-related complications and hypertension. Multidisciplinary teams conduct comprehensive evaluations considering the multifaceted nature of SCA and hypertension.^[27]^ This assessment helps in developing individualized care plans that encompass BP management, SCA-specific treatments, and addressing other comorbidities.

Collaborative efforts enable healthcare providers to coordinate care seamlessly, optimizing treatment strategies while minimizing potential drug interactions and adverse effects. Regular consultations among specialists facilitate adjustments in treatment plans based on evolving patient needs. Multidisciplinary teams emphasize patient education, empowering individuals with SCA and hypertension to actively participate in their care. Providing comprehensive information, and educating patients about treatment goals enhance patient engagement and adherence to management plans.^[27]^ Integrated care models ensure a smooth transition of care across various healthcare settings and specialties. This coordinated approach facilitates communication between healthcare providers during transitions, such as from pediatric to adult care, ensuring continuity and quality of care.^[26]^ Collaborative care models emphasize preventive strategies and routine health maintenance, including regular BP monitoring, screenings for complications, vaccinations, and health promotion activities tailored for SCA patients with hypertension. Multidisciplinary teams often engage in research initiatives and the integration of innovative approaches within clinical practice. This involvement fosters the adoption of novel treatments, technology, and best practices, contributing to advancements in the field.^[25]^ Integrated care models and multidisciplinary approaches serve as a framework for optimizing healthcare delivery to individuals with SCA and hypertension. By leveraging the expertise of diverse healthcare professionals, these models aim to improve patient outcomes, enhance quality of life, and address the multifaceted challenges associated with managing both SCA and hypertension concurrently.

### 3.12. Addressing disparities and global health initiatives

Addressing disparities in healthcare access and implementing global health initiatives are crucial components in improving the management of hypertension in individuals with SCA on a global scale.^[28–30]^ Disparities in healthcare access and quality of care persist in various regions, impacting the diagnosis, treatment, and outcomes of individuals with SCA and hypertension. Disparities in healthcare access affect individuals with SCA and hypertension, particularly in underserved communities or regions with limited resources. Efforts should focus on improving access to specialized care, medications, diagnostic tools, and healthcare facilities for vulnerable populations. Promoting health education and raising awareness about SCA and its complications, including hypertension, is crucial.^[31]^ Educating healthcare providers, patients, families, and communities about the importance of regular screenings, early detection, and adherence to treatment regimens can significantly impact outcomes. Tailoring healthcare services to be culturally sensitive and linguistically appropriate is essential. Investing in healthcare infrastructure, training healthcare professionals, and building capacity in regions lacking resources are vital initiatives. This involves providing education, training programs, and resources to healthcare providers to improve their capabilities in managing SCA and hypertension effectively. Transgender and gender-diverse individuals with SCD may face unique challenges related to stigma, access to care, and the impact of gender-affirming hormone therapy on cardiovascular risk profiles. It is imperative that clinicians adopt inclusive care approaches and recognize the need for individualized hypertension management strategies in this population.

Encouraging research initiatives and data collection efforts aimed at understanding the prevalence, impact, and treatment outcomes of hypertension in individuals with SCA across diverse populations is crucial.^[28]^ This information is instrumental in developing targeted interventions and policies. Advocating for policies that prioritize SCA and hypertension management, ensuring access to essential medications, and supporting funding for research and healthcare programs are essential.^[32]^ Collaborating with policymakers, advocacy groups, and healthcare organizations can drive positive changes in healthcare delivery. International collaboration among healthcare organizations, governmental bodies, nonprofit organizations, and global health initiatives facilitates the sharing of best practices, resources, and expertise. Partnerships foster knowledge exchange and support the implementation of effective strategies worldwide. Addressing disparities and implementing global health initiatives require a multifaceted approach involving stakeholders at various levels. By focusing on improving healthcare access, education, capacity building, and advocacy, efforts can be directed towards reducing the burden of hypertension in individuals living with SCA, ultimately leading to improved health outcomes and quality of life on a global scale.

### 3.13. Recent molecular insights and therapeutic controversies: linking mechanisms to clinical management

Advances in molecular hematology and vascular biology have shed light on the intricate interplay of hemolysis, endothelial injury, and inflammatory activation that underlies hypertension in SCD. Central to these mechanisms is NO depletion, a consequence of chronic hemolysis in which free plasma hemoglobin and heme scavenge NO and release arginase, lowering the availability of its precursor, L-arginine. Reduced NO disrupts endothelial homeostasis, promotes vasoconstriction, and accelerates vascular remodeling – pathways strongly implicated in the development of systemic and pulmonary hypertension. Parallel activation of the endothelin-1 pathway, a potent vasoconstrictor axis, contributes to vascular stiffness and sodium retention, while ROS generated by ischemia–reperfusion cycles amplify oxidative stress and impair vascular compliance. Recent studies also implicate the renin–angiotensin–aldosterone system (RAAS) and renal sodium transporters in maladaptive fluid regulation, highlighting a mechanistic bridge between sickle nephropathy and BP elevation.^[33]^

These molecular insights have direct therapeutic implications but also fuel important clinical debates. For example, ACE inhibitors and ARBs are widely used for microalbuminuria and renal protection, yet concerns remain about their long-term effects on potassium homeostasis and the potential to worsen anemia by lowering erythropoietin activity. Similarly, calcium channel blockers, while effective antihypertensives, have been questioned for their potential to exacerbate peripheral edema in a population already prone to fluid imbalance. Hydroxyurea, the cornerstone of disease modification, indirectly improves vascular tone by reducing hemolysis and increasing fetal hemoglobin, but its impact on BP control remains variable across studies, creating uncertainty regarding its role as a primary antihypertensive adjunct. Novel agents such as endothelin receptor antagonists and NO donors show mechanistic promise but lack robust randomized trial data, leaving their clinical adoption cautious and heterogeneous.^[34,35]^

Connecting these pathways to management underscores the need for a mechanism-driven therapeutic strategy. Endothelial protection through RAAS inhibition addresses renal and vascular targets simultaneously, while antioxidant approaches, including hydroxyurea and emerging heme-scavenging agents, aim to restore NO signaling and reduce oxidative injury. Ambulatory BP monitoring and renal biomarker profiling can help identify patients in whom hemolysis-driven vascular dysfunction predominates, supporting individualized drug selection. Ultimately, these molecular revelations emphasize that hypertension in SCD is not merely a hemodynamic consequence but a reflection of ongoing endothelial and renal pathology. Therapeutic decisions must therefore balance conventional BP control with interventions that interrupt the underlying disease biology, an evolving paradigm that will benefit from future trials integrating mechanistic endpoints with clinical outcomes.^[36]^

## 4. Conclusion

Hypertension in SCD represents more than a simple elevation in BP; it reflects a complex convergence of hemolysis-driven endothelial dysfunction, renal injury, inflammatory activation, and altered vascular tone. Even modest increases in pressure, once considered clinically insignificant, now emerge as potent predictors of stroke, nephropathy, and early mortality. Effective management therefore requires an approach that transcends conventional antihypertensive protocols to incorporate the unique molecular and pathophysiologic features of SCD.

Recent advances in understanding NO depletion, endothelin signaling, and oxidative stress provide a foundation for mechanism-based therapies, while ongoing innovations (including RAAS blockade, hydroxyurea optimization, endothelin antagonists, NO donors, and emerging gene-editing strategies) offer opportunities for targeted intervention. At the same time, controversies surrounding drug selection, optimal BP thresholds, and long-term safety highlight the need for carefully designed clinical trials to guide evidence-based practice. Moving forward, integrating molecular biomarkers with individualized pharmacologic and lifestyle strategies holds the greatest promise for improving outcomes. By combining precise BP control with interventions that address the underlying biology of SCD, clinicians can not only reduce hypertensive complications but also modify the trajectory of end-organ damage, ultimately enhancing survival and quality of life for this high-risk population.

## Author contributions

**Conceptualization:** Emmanuel Ifeanyi Obeagu.

**Methodology:** Emmanuel Ifeanyi Obeagu.

**Supervision:** Emmanuel Ifeanyi Obeagu.

**Validation:** Emmanuel Ifeanyi Obeagu.

**Visualization:** Emmanuel Ifeanyi Obeagu.

**Writing – original draft:** Emmanuel Ifeanyi Obeagu.

**Writing – review & editing:** Emmanuel Ifeanyi Obeagu.
